# Loss of ischemic tolerance with age: can we protect an old kidney

**DOI:** 10.1093/geroni/igab046.2570

**Published:** 2021-12-17

**Authors:** Egor Plotnikov

**Affiliations:** A.N. Belozersky Institute of Physico-Chemical Biology, Lomonosov Moscow State University, Moscow, Moskva, Russia

## Abstract

The most abundant and vulnerable cohort of patients with acute kidney injury (AKI) is represented by the older people. It is well-known, the kidney tissue undergoes some changes with age, both at the morphological and molecular level. Therefore, when treating AKI in older patients, it is necessary to take into account the morphofunctional features of aging kidney tissue and metabolic alterations. We have shown that the kidney of old rats does not perceive signals from the most well-known protective approaches such as ischemic preconditioning (IPC) and caloric restriction (CR). Although the old kidney did not develop more severe AKI after ischemia, we found no pronounced effect on attempts to increase its resistance by IPC and CR. Analysis of the mechanisms underlying this loss of tolerance has shown that the most affected pathways are the mechanism of mitochondrial quality control, the effectiveness of autophagy, and the proliferative potential of kidney cells. However, several protective pathways activated in the young kidney were also active in the old one in response to the CR. In particular, an increase in SIRT1 deacetylase, antiapoptotic Bcl-xL, and a decrease in oxidative stress were observed. Our results show that some defense systems demonstrating their effectiveness in young organisms lose their beneficial effect in old organisms, while others still can be activated by protective approaches. Thus, it is necessary to carefully analyze the possibilities of increasing ischemic tolerance for old organisms. This work was supported by the Russian science foundation (grant #21-75-30009).

